# Dapansutrile mitigates methotrexate-induced hepatotoxicity in rats: roles of inflammation, oxidative stress, pyroptosis, and autophagy

**DOI:** 10.3389/fphar.2026.1798557

**Published:** 2026-06-23

**Authors:** Haider H. Motlak, Mahmoud Elshal, Marwa S. Serrya

**Affiliations:** 1 Department of Pharmacology and Toxicology, Faculty of Pharmacy, Mansoura University, Mansoura, Egypt; 2 AlGhad College for Applied Medical Sciences, Najran, Saudi Arabia

**Keywords:** dapansutrile, hepatotoxicity, methotrexate, oxidative stress and inflammation, pyroptosis, autophagy

## Abstract

**Aims:**

Methotrexate (MTX) is a widely used chemotherapeutic and immunosuppressive agent; however, its clinical utility is frequently limited by dose-dependent hepatotoxicity which was mediated through oxidative stress and dysregulated inflammatory signaling. The present study investigated the protective effect of dapansutrile (DAPA), a selective NLRP3 inflammasome inhibitor, against MTX-induced hepatic injury in rats and elucidated the underlying molecular mechanisms.

**Methods:**

MTX hepatotoxicity was induced by a single intraperitoneal injection (20 mg/kg), while DAPA was administered orally at doses of 10 or 20 mg/kg for seven consecutive days.

**Results:**

MTX administration resulted in pronounced liver dysfunction, as evidenced by marked elevations in serum ALT, AST, ALP, and GGT levels, severe histopathological alterations, enhanced lipid peroxidation, depletion of endogenous antioxidants, and activation of TLR4/MyD88/NF-κB signaling. Furthermore, MTX robustly triggered NLRP3 inflammasome activation, leading to increased caspase-1 activity, elevated IL-1β and IL-18 levels, enhanced gasdermin D cleavage, and induction of pyroptotic cell death. MTX also disrupted hepatic autophagic activity, as indicated by reduced LC3-II levels and p62 accumulation. DAPA treatment significantly ameliorated these biochemical, histological, and molecular abnormalities. DAPA suppressed oxidative stress, attenuated inflammatory cytokine production, inhibited inflammasome-mediated pyroptosis, and restored autophagic balance.

**Conclusion:**

Collectively, these findings demonstrate that DAPA confers robust hepatoprotection against MTX-induced toxicity through coordinated modulation of inflammatory, oxidative, pyroptotic, and autophagic pathways.

## 1 Introduction

Drug-induced organ toxicity represents one of the main challenges in clinical practice, and thus it limits the use of many effective therapeutic agents. When it comes to chemotherapeutic agents, they are often associated with off-target toxicities and could lead to vital organ dysfunction and thus adversely impact the patient’s quality of life (Peter Guengerich, 2011; Zeien et al., 2022). Methotrexate (MTX) is a widely used chemotherapeutic and immunosuppressive agent. However, its clinical utility is frequently limited by dose-dependent hepatotoxicity. This toxicity is mediated through oxidative stress and dysregulated inflammatory signaling (Hamed et al., 2022; Zarén et al., 2023).

The liver is highly susceptible to MTX-induced toxicity, as MTX disrupts redox homeostasis by increasing ROS generation and weakening antioxidant defenses, leading to oxidative stress, lipid peroxidation, mitochondrial dysfunction, and hepatocellular injury (Mehrzadi et al., 2018; El-Sheikh et al., 2015). At the same time, MTX could activate some key signaling pathways, such as nuclear factor-kappa B (NF-κB) and Toll-like receptor 4 (TLR4)/myeloid differentiation primary response 88 (MyD88), and initiate inflammatory cascades by stimulating the release of pro-inflammatory cytokines. All these processes together would aggravate hepatic tissue injury and lead to progressive liver dysfunction (Matouk et al., 2022).

Given the fact that oxidative stress and inflammation play a crucial role in MTX-induced hepatotoxicity, there has been a large interest in targeting these mechanisms to protect against hepatic injury induced by MTX. Dapansutrile (DAPA, OLT1177), which is a selective inhibitor of the NOD-like receptor family pyrin domain-containing 3 (NLRP3) inflammasome, has been studied as a promising anti-inflammatory agent that can offer some therapeutic potential (Kiran et al., 2022). When the NLRP3 inflammasome is activated, this will lead to the cleavage of caspase-1 and subsequently the maturation of inflammatory cytokines, including IL-1β and IL-18. Drug-induced liver injury is reported to dysregulate the NLRP3/caspase-1 axis just like many inflammatory and degenerative diseases (Swanson et al., 2019).

DAPA specifically inhibits the NLRP3 inflammasome activation without suppressing a broader innate immune response. This activity makes DAPA a promising therapeutic candidate, with preclinical studies demonstrating its antioxidant, anti-inflammatory, and tissue-protective effects (Kiran et al., 2022; Marchetti et al., 2018). DAPA’s ability to be given orally along with its good safety profile can support its potential use in hepatotoxicity induced by certain chemotherapeutic agents such as MTX. Accordingly, the present study was designed to evaluate the protective effect of DAPA on MTX-induced hepatotoxicity in rats and shed some light on the potential underlying mechanisms.

## 2 Materials and methods

### 2.1 Animals

Adult males of Sprague-Dawley rats weighing on average 170–230 g were obtained from VACSERA (Giza, Egypt). The rats were put under standard conditions which are 25 °C ± 2 °C and 12 h light/dark cycles allowing free access to food and water. All of the procedures were approved by Mansoura University Animal Care and Use Committee (MU-ACUC Protocol No.: PHARM. MS.24.09.114).

### 2.2 Drugs and chemicals

MTX (Unitrexate® 50 mg/2 mL) was obtained from Hikma Pharmaceuticals (Amman, Jordan). DAPA powder was purchased from HEOWNS (Tianjin, China) and freshly dissolved in 0.9% normal saline prior to administration. Secobarbital sodium was obtained from Sigma-Aldrich (MA, USA) was dissolved in normal saline and used for anesthesia. All other reagents were of best analytical grade. All other kits and reagents details are provided in the Supplementary Material (Supplementary Tables S1-S3).

### 2.3 Experimental design

MTX hepatotoxicity was induced by a single intraperitoneal (i.p.) injection of MTX at a dose of 20 mg/kg (Akman et al., 2023). On the other hand, DAPA at a dose of 10 or 20 mg/kg was administered once orally daily for seven consecutive days (Saber et al., 2021). Rats were randomly divided into five groups (n = 6 per group) as follows: *(1) CTRL (Control) group:* received oral 0.9% normal saline once daily for seven consecutive days and i. p. Injected with 0.9% normal saline on the second day, *(2) DAPA group:* received DAPA (20 mg/kg, orally) once daily for 7 days and i. p. Injected with 0.9% normal saline on the second day, *(3) MTX group:* received oral 0.9% normal saline daily for seven consecutive days and injected with MTX (20 mg/kg, i. p.) on the second day, *(4) DAPA10 + MTX:* received oral DAPA at a dose of 10 mg/kg, daily for seven consecutive day and MTX (20 mg/kg, i. p.) on the second day, and *(5) DAPA20 + MTX:* received DAPA (20 mg/kg) and MTX as described above. On the eighth day, animals were anesthetized with secobarbital (50 mg/kg, i. p.) before euthanized by cervical dislocation.

### 2.4 Sample collection and preparation

Blood sample (about 3 mL) were withdrawn from the retro-orbital plexus under anesthesia. The sample was left aside to clot and then was centrifuged at room temperature at a speed of 3,000 rpm for 15 min. Immediately after centrifugation, the obtained serum samples were used for the biochemical analysis of liver enzyme. The livers were excised then rinsed with cold normal saline. One part was fixed in 10% neutral-buffered formalin for histopathological and immunohistochemical analysis. Another part was divided into three equal slices and stored at − 80 °C for further ELISA, PCR, and Western blot analyses.

### 2.5 Liver function biomarkers

Alanine aminotransferase (ALT), aspartate aminotransferase (AST), alkaline phosphatase (ALP), and gamma-glutamyl transferase (GGT) serum activities were evaluated using colorimetric assay kits (BioMed, Egypt). All enzyme activities were expressed in units per liter (U/L) according to the calibration factors provided by the manufacturers.

### 2.6 Histopathological evaluation

Formalin-fixed liver tissues were paraffin-embedded and sectioned at a width of 5 μm slices, followed by staining with hematoxylin and eosin stains (H&E). Sections were examined microscopically at magnification powers of ×100 and ×400 magnification powers. The histopathological examination was concentrated on the presence and severity of the following hepatic changes which are: hepatocyte degeneration, hepatocyte necrosis, portal inflammation, lobular inflammatory foci, and vascular dilation/congestion (Leow et al., 2023). Each criterion was graded on a semiquantitative scale ranging from 0 to 3 as following: 0 = absent; 1 = mild; 2 = moderate; 3 = severe. Scoring was rated by an experienced pathologist who was blinded to groups’ identity.

### 2.7 Immunohistochemical analysis

The expression of NF-κB p65 and active caspase-1 was assessed through immunohistochemical staining utilizing paraffin-embedded liver sections of 4-5 µm thickness. The sections were deparaffinized in xylene and then rehydrated with graded ethanol of different concentrations (100%, 95%, and 75%). Subsequently, the sections are subjected to heat-induced epitope retrieval (HIER) using a citrate buffer at pH 6.0 and a pressure cooker (Cell Marque Trilogy system). Certain quantities of 3% hydrogen peroxide solution were also utilized to inhibit endogenous peroxidase activity. Incubation of the tissue sections was followed using primary antibodies (rabbit polyclonal anti-NF-κB p65 and rabbit polyclonal anti-caspase-1). Washing was done on the slides and then incubated with Ultravision One HRP polymer (Thermo Fisher Scientific) for a period of 15 min. Diaminobenzidine substrate solution was used to make the picture. Mayer’s hematoxylin was used to counterstain (Ramos-Vara, 2005). Finally, the sections were dried out by using increasing amount of alcohol, cleared in xylene, and put on coverslips. Images were taken at 100x and 400x magnifications and percentage expression of NF-κB p65 and active caspase-1 was determined.

### 2.8 Hepatic oxidative stress and antioxidant parameters

Malondialdehyde (MDA), reduced glutathione (GSH), and total antioxidant capacity (TAC) were quantified in liver tissue homogenates using colorimetric assay kits from Bio-Diagnostic (Giza, Egypt), following the manufacturer’s protocols. Liver tissue was homogenized as a 10% (w/v) solution (0.1 g tissue in 1 mL phosphate-buffered saline). Concentrations were determined from standard curves and expressed as nmol/mL of homogenate. Values were then normalized to tissue weight; since 100 mg tissue was homogenized in 1 mL, nmol/mL is directly equivalent to nmol/100 mg tissue. Accordingly, all results are expressed as nmol/100 mg tissue.

### 2.9 Enzyme-linked immunosorbent assay (ELISA)

Hepatic levels of Toll-like receptor 4 (TLR4), myeloid differentiation primary response 88 (MyD88), tumor necrosis factor-alpha (TNF-α), interleukin (IL)-6, IL-1β, IL-18, NLRP3, apoptosis-associated speck-like protein containing a CARD (ASC), and gasdermin D-N-terminal (GSDMD-N) were measured using ELISA kits. Samples were processed in accordance with the respective manufacturer’s instructions.

### 2.10 Western blot analysis

Liver tissue was checked for protein expression of the autophagy-related markers which are autophagy-related protein light chain 3 (LC3)-II and p62. The tissues were homogenized in RIPA buffer containing protease inhibitors. Then, protein concentration was calculated using the Bradford assay. Equal amounts of protein were run onto SDS-PAGE and the gel was then transferred onto PVDF membranes. The PVDF membranes incubated overnight at 4 °C with rabbit polyclonal primary antibodies against LC3A/LC3B and p62 after proper blocking of the membranes. The membranes were then washed with buffer and incubated with HRP-conjugated secondary antibodies. Finally, protein bands were visualized using enhanced chemiluminescence (ECL). Band intensities were assessed using appropriate software where the β-actin was used as the internal loading control.

### 2.11 Quantitative real-time PCR (qRT-PCR)

Liver tissues were checked for mRNA expression of NLRP3, NF-κB, and GSDMD genes. Liver specimens were used from which total RNA are extracted using the Total RNA Isolation System (Thermo Scientific, USA) following the instructions of the kit provider carefully. The purity and concentration of the extracted RNA was determined using a UV-Visible spectrophotometry at wavelength of 260 nm. High-Capacity cDNA Reverse Transcription Kit (Thermo Fisher Scientific) was used to generate cDNA from the total RNA using a quantity of 1 μg of this RNA. Maxima SYBR Green qPCR Master Mix (Thermo Fisher Scientific, USA) and Stepone™ Real-time PCR machine (Applied Biosystems) were utilized in this analysis. Each PCR reaction mixture (25 μL) contained 12.5 μL of 2x SYBR Green Master Mix, 0.3 μM of each primer, and 500 ng cDNA. The amplification program consisted of the following steps: initial denaturation at 95 °C for 10 min, 40 cycles of 95 °C for 15 s, and 60 °C for 60 s. The relative expression levels of NLRP3, NF-κB, and GSDMD genes were normalized to β-actin (being the housekeeping gene). Value changes were then calculated using the 2^−ΔΔCT^ method. Primer sequences used are provided in Supplementary Table S4 the supplementary data.

### 2.12 Statistical analysis

Data are expressed as mean ± SEM. Group comparisons were primarily performed using one-way ANOVA followed by Tukey’s *post hoc* test. Histopathological semi-quantitative lesion scores were analyzed using the Kruskal–Wallis test with Dunn’s multiple comparison procedure. P < 0.05 was considered statistically significant. All statistical analyses and graphs were generated using GraphPad Prism 9.0 (GraphPad Software, San Diego, CA, USA).

## 3 Results

### 3.1 Effect of DAPA on hepatic function biomarkers in MTX-intoxicated rats

**FIGURE 1:**
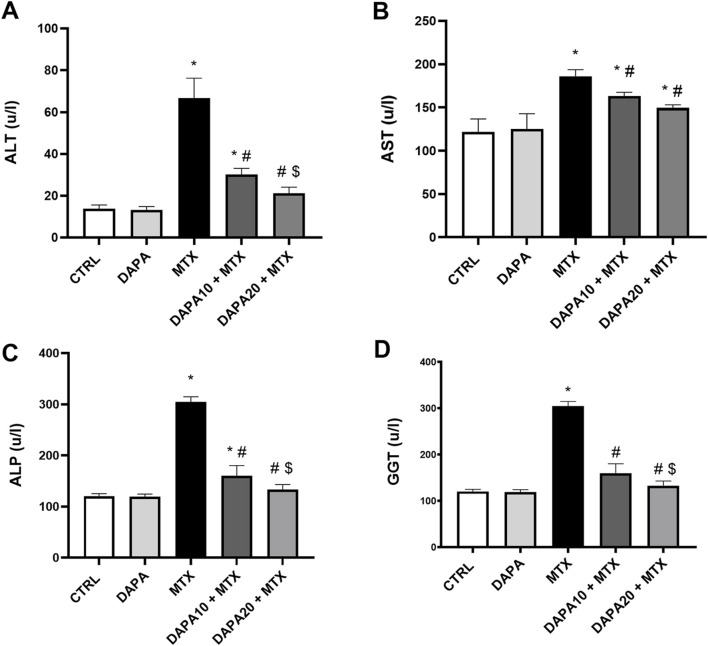
Effect of DAPA (dapansutrile) on hepatic enzyme levels in MTX (methotrexate)-induced hepatotoxicity in rats. **(A)** ALT (alanine aminotransferase), **(B)** AST (aspartate aminotransferase), **(C)** ALP (alkaline phosphatase), and **(D)** GGT (gamma-glutamyl transferase). Data are expressed as mean ± SEM (n = 6). ^*, #, $^ significant difference compared to the CTRL (control), MTX, and DAPA10 + MTX groups, respectively.

MTX-intoxication resulted in significant hepatocellular damage evidenced by marked increases in liver enzyme levels in the serum. As illustrated in Figure 1A, serum ALT exhibited the most pronounced increase (4.84-fold) compared to the CTRL group. AST levels were also significantly higher, going up by about 2.2 times (Figure 1B). MTX also caused Cholestatic and biliary injury, as indicated by significant elevations in ALP by 2.01-fold (Figure 1C) and GGT by 1.90-fold (Figure 1D).

Otherwise, DAPA intervention significantly and in a dose-related manner reduced these biochemical abnormalities. DAPA (10 mg/kg) lowered ALT by 54.8%, AST by 22.2%, ALP by 42.8%, and GGT by 47.5%. At the higher dose of 20 mg/kg, more substantial reductions were observed; ALT went down by 68.2%, AST by 39.3%, ALP by 47.4%, and GGT by 56.3%.

### 3.2 Effect of DAPA on histopathological abnormalities of liver tissue in MTX-intoxicated rats

**FIGURE 2:**
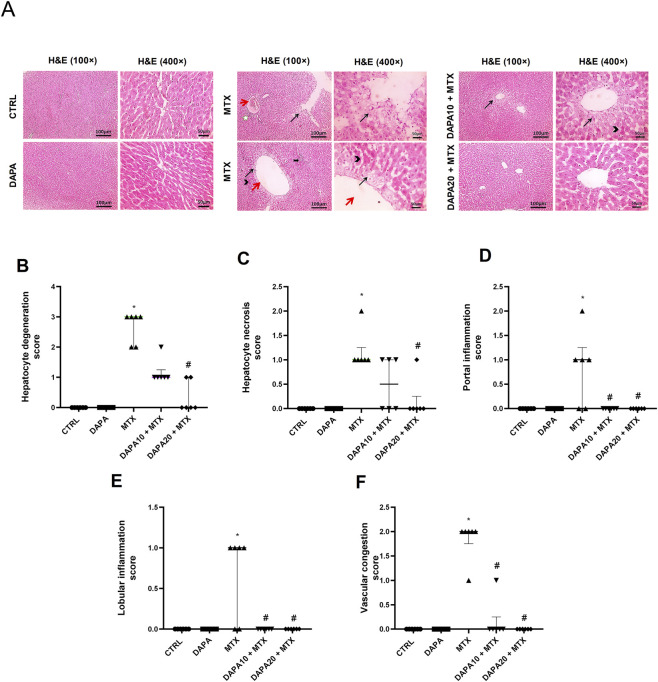
Effect of DAPA (dapansutrile) on hepatic histopathological lesions and corresponding semi-quantitative scores in MTX (methotrexate)-intoxicated rats. **(A)** Representative H&E-stained liver sections (100× and 400×) from the CTRL (control), DAPA-only, MTX, DAPA10 + MTX, and DAPA20+MTX groups, **(B)** Hepatocyte degeneration score, **(C)** Hepatocyte necrosis score, **(D)** Portal inflammation score, **(E)** Lobular inflammation score, and **(F)** Vascular congestion score.

Microscopic analysis of H&E-stained liver sections demonstrated distinct variations in hepatic architecture among the experimental groups (Figure 2A). The CTRL group exhibited a typical histological pattern, defined by radially arranged hepatic cords surrounding the central veins, preserved portal areas, and normal sinusoidal spaces. The DAPA-only group displayed similar morphology, with well-preserved hepatocytes and normal lobular architecture, which showed that the compound was safe for the liver.

Conversely, liver sections from the MTX-injected group exhibited significant pathological changes, which include vascular dilation and congestion (red arrows), perivascular hydropic degeneration of hepatocytes (thin black arrows), focal hepatocellular necrosis (black arrowheads), portal inflammation (thick white arrow), and focal lobular inflammatory foci (thick black arrow). These significant morphological abnormalities indicate the established hepatotoxic characteristics of MTX, which include oxidative damage, inflammation, and initial necroinflammatory processes.

Alternatively, treatment with DAPA (10 mg/kg) partially improved the lesions caused by MTX, showing centrilobular hydropic degeneration (thin black arrows) and fewer necrotic hepatocytes (black arrowheads) than the MTX group. Otherwise, the protective effect was more pronounced in the DAPA (20 mg/kg)-treated group, which demonstrated a largely preserved hepatic architecture with normal-appearing hepatocytes, central veins, portal areas, and sinusoids, closely resembling the normal histology.

Semi-quantitative scoring supported these microscopic findings (Figures 2B–F). MTX significantly increased hepatocyte degeneration, hepatocyte necrosis, portal inflammation, lobular inflammation, and vascular congestion compared with the CTRL group. Both DAPA treatment groups showed dose-related reductions in all lesion scores, with the higher dose restoring most parameters toward the normal values.

### 3.3 Effect of DAPA on hepatic oxidative stress biomarkers in MTX-intoxicated rats

**FIGURE 3:**
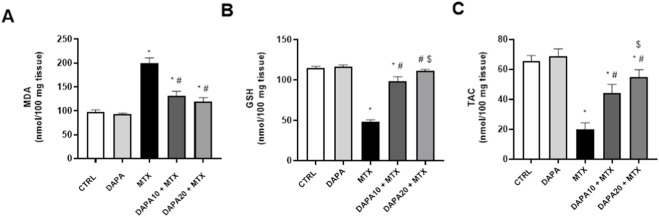
Effect of DAPA (dapansutrile) on hepatic oxidative stress biomarkers in MTX (methotrexate)-intoxicated rats. **(A)** MDA (malodialdehyde), **(B)** GSH (reduced glutathione), and **(C)** TAC (total antioxidant capacity). Data are expressed as mean ± SEM (n = 6). ^*, #, $^ significant difference compared to the CTRL (control), MTX, and DAPA10 + MTX groups, respectively.

MTX induced profound oxidative stress, as indicated by about 2.1-fold higher hepatic MDA levels (Figure 3A), confirming enhanced lipid peroxidation. Furthermore, MTX significantly depleted endogenous antioxidant defenses: GSH and TAC levels decreased by 58.4% and 69.8%, respectively (Figures 3B,C, respectively). On the other hand, DAPA exerted a strong antioxidant effect at both doses. At 10 mg/kg, MDA decreased by 34.1%, while GSH and TAC increased by about 2-fold and 2.2-fold, respectively. At 20 mg/kg, MDA was further reduced by 40%, and GSH and TAC were elevated by about 2.3-fold and 2.8-fold.

### 3.4 Effect of DAPA on TLR4/MyD88 signaling in MTX-intoxicated rats

**FIGURE 4:**
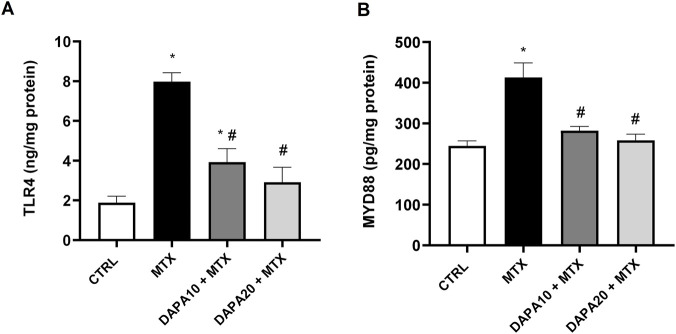
Effect of DAPA (dapansutrile) on TLR4 and MyD88 levels in liver tissue in MTX (methotrexate)-intoxicated rats. **(A)** TLR4 (Toll-like receptor 4) and **(B)** MyD88 (myeloid differentiation primary response 88). Data are expressed as mean ± SEM (n = 6). ^*, #^ significant difference compared to the CTRL (control) and MTX groups, respectively.

MTX significantly activated upstream inflammatory signaling pathways. As shown in Figure 4A, MTX injection significantly increased TLR4 protein levels by 4.2-fold compared to the CTRL group. This was accompanied by a 1.68-fold elevation in MyD88 protein levels (Figure 4B). Meanwhile, DAPA intervention significantly reduced both mediators. At 10 mg/kg, TLR4 and MyD88 decreased by 50.7% and 31.7%, respectively. At 20 mg/kg, reductions reached 63.5% and 37.6%, respectively.

### 3.5 Effect of DAPA on hepatic NF-κB p65 expression in MTX-intoxicated rats

**FIGURE 5:**
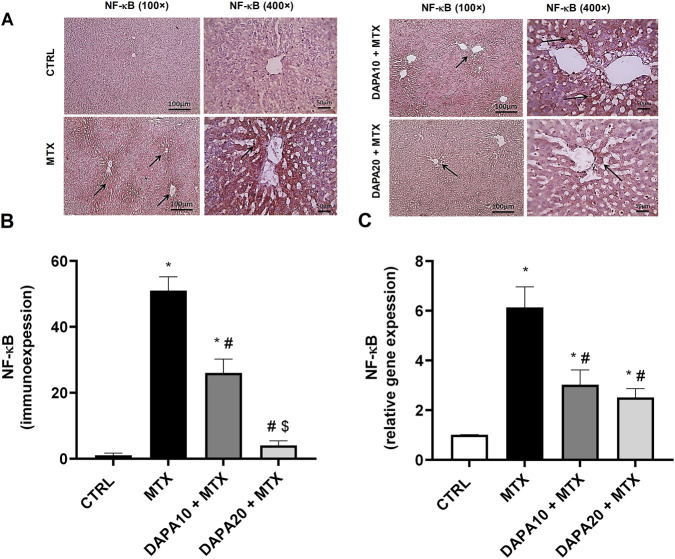
Effect of DAPA (dapansutrile) on NF-κB p65 (Nuclear factor kappa-B p65) hepatic expression in MTX (methotrexate)-intoxicated rats. **(A)** Representative immunohistochemistry images (100× and 400×), **(B)** Corresponding immunoexpression percentages, and **(C)** NF-κB gene expression levels. Data are expressed as mean ± SEM (n = 6). ^*, #, $^ significant difference compared to the CTRL (control), MTX, and DAPA10 + MTX groups, respectively.

Immunohistochemical staining of NF-κB p65 showed clear differences between the experimental groups (Figure 5A). The CTRL group exhibited negative NF-κB staining, with hepatocytes displaying normal morphology and only subtle background coloration. Likewise, the DAPA-only group exhibited no discernible NF-κB activation, thereby validating the lack of inflammatory induction by the drug.

In stark contrast, liver sections from the MTX-injected group exhibited pronounced positive brown cytoplasmic NF-κB staining in numerous hepatocytes (black arrows). Numerous stained cells were clustered around central veins and portal areas, and the intense brown immunoreactivity correlated with areas where the architecture was distorted and inflammatory cells were present. This strong staining shows that NF-κB is very active because of oxidative and inflammatory stress caused by MTX. Otherwise, treatment with DAPA led to a dose-related decrease in NF-κB expression. The DAPA10 + MTX group showed less brown staining (black arrows), which means that NF-κB activation was only partially blocked. Otherwise, in the DAPA20 + MTX group, NF-κB staining was mild and dispersed, with sporadic hepatocytes exhibiting light brown cytoplasmic positivity (black arrows), resembling a near-normal histological appearance.

Furthermore, quantitative analysis corroborated these findings (Figure 5B). MTX significantly elevated NF-κB immunoexpression, while DAPA treatment diminished NF-κB expression percentage in a graded fashion, with the 20 mg/kg dose demonstrating the most pronounced suppression.

In addition, the histological results matched up with the gene expression data (Figure 5C). MTX increased NF-κB mRNA expression by about 6.1 times. Meanwhile, DAPA intervention significantly inhibited NF-κB transcription, decreasing mRNA levels by 50.7% at 10 mg/kg and 59.1% at 20 mg/kg. Together, these results demonstrate that DAPA effectively counteracts MTX-induced NF-κB activation, limiting inflammatory signaling through strong inhibition of both NF-κB protein expression and gene transcription.

### 3.6 Effect of DAPA on TNF-α and IL-6 hepatic expression levels in MTX-intoxicated rats

**FIGURE 6:**
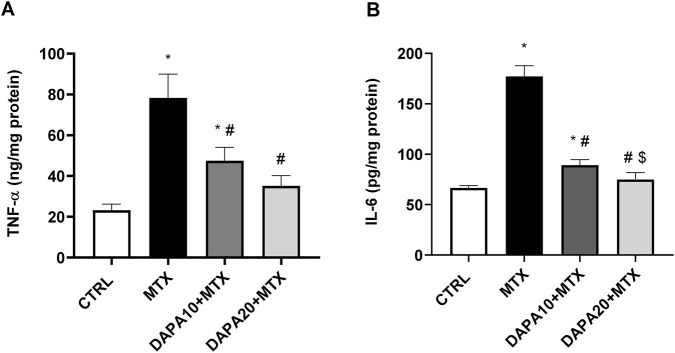
Effect of DAPA (dapansutrile) on Pro-inflammatory cytokine hepatic levels in MTX (methotrexate)-intoxicated rats. **(A)** TNF-α (tumor necrosis factor-alpha) and **(B)** IL-6 (interleukin-6) protein levels. Data are expressed as mean ± SEM (n = 6). ^*, #, $^ significant difference compared to the CTRL (control), MTX, and DAPA10 + MTX groups, respectively.

MTX significantly elevated hepatic TNF-α and IL-6 levels (Figures 6A,B, respectively), consistent with its known ability to activate cytokine-driven inflammatory injury. TNF-α levels increased more than 3.3-fold, while IL-6 levels increased by about 2.7-fold, highlighting the intense inflammatory pressure imposed on liver tissue. Alternatively, DAPA intervention produced a substantial decrease in cytokine expression. At the dose of 10 mg/kg, TNF-α and IL-6 levels were reduced by 39.3% and 49.7%, respectively. The 20 mg/kg dose exerted stronger suppression (55.1% and 57.6% reductions, respectively) nearly halving the MTX-induced cytokine storm.

### 3.7 Effect of DAPA on NLRP3 inflammasome activation in MTX-intoxicated rats

**FIGURE 7:**
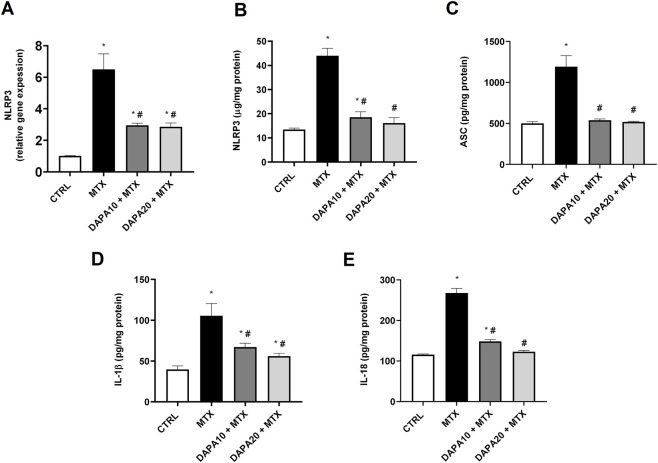
Effect of DAPA (dapansutrile) on hepatic NLRP3 (NOD-like receptor family pyrin domain-containing 3) inflammasome components in MTX (methotrexate)-intoxicated rats. **(A)** NLRP3 mRNA, **(B)** NLRP3 protein, **(C)** ASC (apoptosis-associated speck-like protein containing a CARD), **(D)** IL (interleukin)-1β, and **(E)** IL-18. Data are expressed as mean ± SEM (n = 6). *, # significant difference compared to the CTRL (control) and MTX groups, respectively.

MTX injection triggered a robust activation of the canonical NLRP3 inflammasome pathway at both the gene and protein levels. qRT-PCR analysis showed that MTX markedly increased NLRP3 mRNA expression by about 6.4-fold compared to the CTRL group (Figure 7A). This transcriptional upregulation was accompanied by a significant elevation in the NLRP3 protein levels, which rose 3.3-fold compared in the MTX-injected rats compared to the control rats (Figure 7B). Downstream inflammasome components were also significantly elevated following the MTX challenge: ASC increased 2.4-fold, IL-1β increased 2.7-fold, and IL-18 increased 2.3-fold (Figures 7C–E). These collective increases confirm strong activation of the NLRP3 signaling and indicate a shift toward pyroptotic inflammatory cell death.

On the other hand, DAPA intervention markedly attenuated this inflammasome activation. At 10 mg/kg, DAPA reduced NLRP3 protein by 57.9%, ASC by 54.8%, IL-1β by 36.2%, and IL-18 by 44.6% relative to the MTX group. At 20 mg/kg, stronger inhibition was observed, lowering NLRP3 by 63.3%, ASC by 56.5%, IL-1β by 46.9%, and IL-18 by 54.1%. These protein-level reductions paralleled significant declines in NLRP3 gene expression, where DAPA decreased NLRP3 mRNA by about 54.7% at 10 mg/kg and 56.1% at 20 mg/kg. These findings further illustrate that DAPA effectively blocks MTX-induced inflammasome priming at the transcriptional level while simultaneously suppressing downstream activation.

### 3.8 Effect of DAPA on active caspase-1 hepatic expression in MTX-intoxicated rats

**FIGURE 8:**
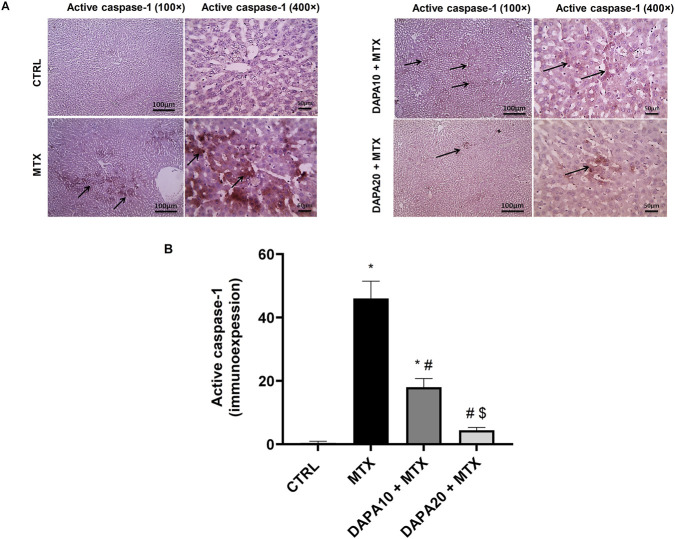
Effect of DAPA (dapansutrile) on active caspase-1 expression in MTX (methotrexate)-intoxicated rats. **(A)** Representative immunohistochemistry images (100× and 400×) and **(B)** Corresponding immunoexpression percentages. Data are expressed as mean ± SEM (n = 6). ^*, #, $^ significant difference compared to the CTRL (control), MTX, and DAPA10 + MTX groups, respectively.

MTX markedly activated caspase-1 that is an important part of the canonical inflammasome-driven pyroptosis process. Figure 8A shows that liver sections from the MTX group exhibit a dense brown staining in the cytoplasm meaning that the caspase-1 activation was widely distributed throughout hepatocytes. Numerous hepatocytes displayed ballooning degeneration and inflammatory infiltrates indicating pyroptotic injury. On the other hand, the quantitative analysis revealed a significant elevation in active caspase-1 immunoexpression (Figure 8B).

The treatment with DAPA dramatically stopped the activation of caspase-1. The 10 mg/kg dose significantly reduced the staining intensity and the immunoexpression. Moreover, the 20 mg/kg dose nearly terminated caspase-1 activation sending expression back to levels same as that to the CTRL group. This pattern demonstrates that the suppression of pyroptotic signaling occurs upstream of IL-1β maturation, indicating that DAPA effectively disrupts the inflammasome–caspase-1 axis.

### 3.9 Effect of DAPA on GSDMD hepatic expression in MTX-intoxicated rats

**FIGURE 9:**
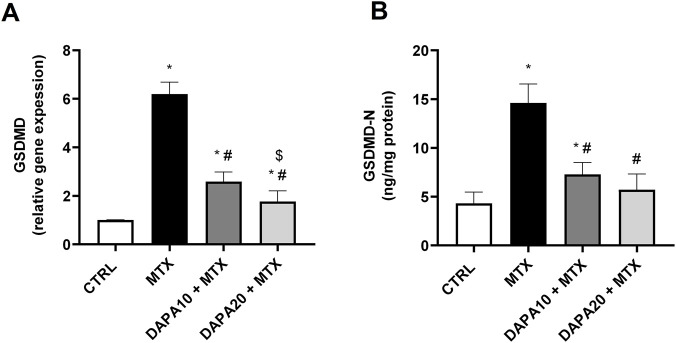
Effect of DAPA (dapansutrile) on hepatic GSDMD (gasdermin D) expression in MTX (methotrexate)-intoxicated rats. **(A)** mRNA and **(B)** protein levels. Data are expressed as mean ± SEM (n = 6). ^*, #, $^ significant difference compared to the CTRL (control), MTX, and DAPA10 + MTX groups, respectively.

MTX injection resulted in a significant activation of pyroptotic execution pathways as demonstrated by marked increases in both GSDMD gene and protein expressions. Results of qRT-PCR analysis demonstrated that the GSDMD mRNA level was about 6.2 times higher than that of the CTRL group (Figure 9A). This increase in transcription can also be seen at the protein level, where GSDMD-N increased by about 3.4 times compared to the CTRL group (Figure 9B). These results confirm that MTX not only activates caspase-1 (the pyroptosis initiator) but also pushes its downstream execution phase, namely, the cleavage of full-length GSDMD into its pore-forming N-terminal fragment, thereby facilitating membrane rupture and pyroptotic cell death.

DAPA significantly inhibited the formation of GSDMD-N in a dose-related manner. At a dose 10 mg/kg, DAPA reduced GSDMD mRNA expression by 58.3% and protein expression levels by approximately 55% compared to the MTX group, emphasizing a significant reduction in pyroptotic drive. The higher dose (20 mg/kg) yielded more pronounced effects by lowering GSDMD mRNA by 71.5% and by lowering also GSDMD-N protein expression levels by approximately 68% compared to the MTX group. This can further confirm the potent inhibitory effect of DAPA on pyroptotic signaling.

### 3.10 Autophagy modulation: LC3-II and p62 expression

**FIGURE 10:**
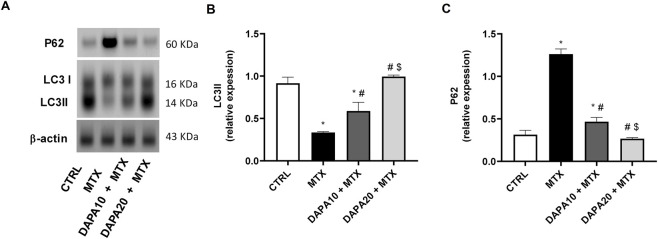
Effect of DAPA (dapansutrile) on autophagy-related protein hepatic levels in MTX (methotrexate)-intoxicated rats. **(A)** Western blot bands, **(B)** autophagy-related protein light chain 3 (LC3)-II expression, and **(C)** p62 expression. Data are expressed as mean ± SEM (n = 3). ^*, #, $^ significant difference compared to the CTRL (control), MTX, and DAPA10 + MTX groups, respectively.

MTX significantly disrupted hepatic autophagy, evidenced by a 66% decrease in LC3-II levels (Figure 10B) and a notable 5.6-fold increase in p62 accumulation (Figure 10C). Western blot analysis (Figure 10A) confirmed this disruption, as evidenced by a reduction in LC3-II conversion and accumulation of p62. DAPA greatly improved the activity of autophagy. At 10 mg/kg, LC3-II levels rose by more than two times, and p62 levels fell by more than 53% compared to the MTX group. These findings may suggest a restoration of autophagy-related processes and an improved autophagic activity. The 20 mg/kg dose led to even more recovery, with LC3-II going up 2.6 times and p62 going down by about 73.1% compared to the MTX group.

## 4 Discussion

MTX is widely used in chemotherapy and immunosuppressive therapy, however, its clinical use is limited by hepatotoxicity. In the present study, the biochemical and histopathological abnormalities characteristic of MTX-induced hepatotoxicity were successfully reproduced through the used animal’s model. The large increases in ALT, AST, ALP, and GGT, together with the extensive hepatocellular degeneration and necroinflammation, are closely similar to the pathological profile reported in previous work where MTX toxicity was characterized as a multifactorial process involving oxidative damage, mitochondrial dysfunction, and dysregulated inflammatory pathways (Ezhilarasan, 2021; Schmidt et al., 2022). The substantial elevation in MDA levels and depletion of endogenous antioxidant defenses, such as GSH and TAC, further confirm the view in which oxidative imbalance plays a crucial role in sensitizing hepatocytes to MTX-mediated stress.

A second distinguished mechanistic feature from our findings is that there was a marked activation of innate immune signaling in general. MTX intoxication notably induced the TLR4/MyD88 axis in accordance with previous reports showing that MTX triggers pattern-recognition receptors, subsequently activating downstream inflammatory mediators (Sukhotnik et al., 2014). In addition, the increase of NF-κB p65 at both the transcriptional and protein levels underlines the major involvement of this transcription factor in amplifying pro-inflammatory cytokine production and making the hepatocytes vulnerable for subsequent inflammatory responses. As described in related models, NF-κB activation promotes the transcription of cytokines such as TNF-α and IL-6, which spread liver injury while simultaneously priming the inflammasome machinery for activation (Bonizzi and Karin, 2004; Taru et al., 2024).

Consistent with this inflammatory priming, our data showed marked activation of the NLRP3 inflammasome in MTX-treated animals, evidenced by increased NLRP3 and ASC expression, caspase-1 activation, and elevated IL-1β and IL-18 levels, all hallmark features of inflammasome activation. It is interesting that these findings are in strong agreement with emerging studies suggesting that MTX can activate NLRP3 through mitochondrial ROS generation, lysosomal destabilization, and ATP release (Mahmoud et al., 2019; El-Sayed et al., 2025). The sharp increase in GSDMD-N, the active cleavage product responsible for pore formation and pyroptotic execution, further supports the idea that pyroptosis is one of the dominant modes of cell death under MTX toxicity. Previous investigations have similarly highlighted the contribution of pyroptosis to liver injury across various pathological conditions, including ischemia-reperfusion injury and chemotherapeutic insult (Alsaab et al., 2025; Wu et al., 2019).

Another significant finding in the current work is that the autophagic activity in MTX-challenged animals was considerably impaired. The decline in LC3-II levels accompanied by substantial p62 accumulation indicates disrupted autophagic flux rather than inhibition of autophagy initiation alone. Autophagy plays a crucial role in removing dysfunctional mitochondria and damaged intracellular components, and its impairment, therefore, amplify an environment where ROS can accumulate and further promote inflammasome activation (Wang et al., 2024; Ali et al., 2025). These outcomes are consistent with previous studies showing that MTX suppresses autophagy in hepatocytes and immune cells and thus worsens tissue damage. The mutual regulation between autophagy and inflammasome signaling has been extensively described, with evidence showing that autophagy deficiency prolongs inflammasome activity and worsens sterile inflammation (Xiong et al., 2020; Sun et al., 2017).

Treatment with DAPA, which is a selective inhibitor of the NLRP3 inflammasome, produced marked hepatoprotection throughout all the studied parameters. Both of the two doses used in this study (10 mg/kg and 20 mg/kg) greatly reduced the biochemical disturbances and the induced histopathological damage, with the higher dose showing a more noticeable improvement. Interestingly, in addition to suppressing the NLRP3 activity, DAPA also appeared to attenuate the upstream mediators TLR4 and MyD88. In spite of the fact that DAPA is traditionally categorized as a direct inflammasome inhibitor, this upstream effect could be explained by disruption of cytokine-driven positive-feedback loops, particularly those involving IL-1β. IL-1β has been reported to actively upregulate pattern recognition receptors like TLR4 and induce TLR4/NF-κB-mediated inflammatory responses through autocrine/paracrine signaling. This phenomenon was associated with other inflammasome-targeting agents as well. Correspondingly, the reductions in NF-κB p65 activity and downstream pro-inflammatory cytokines (TNF-α, IL-6, IL-1β, and IL-18) observed in this study further strengthen the hypothesis that inhibiting NLRP3 could produce a broad anti-inflammatory “ripple effect” (Petrascu et al., 2025; Su et al., 2005).

DAPA’s most distinguished benefit was its potent suppression of pyroptosis. The marked decrease in active caspase-1 immunoreactivity and GSDMD-N expression clearly demonstrates the ability of this compound to interrupt the terminal cell-rupturing phase of inflammatory cell death. This is considered highly relevant provided the mounting evidence that pyroptosis is a central pathological mechanism in drug-induced hepatic injury such as MTX toxicity (Alsaab et al., 2025; Yang et al., 2025). By inhibiting pyroptotic performance, DAPA provides a mechanistically precise mode of cytoprotection that cannot be offered by other conventional antioxidants or broad-spectrum anti-inflammatory compounds.

Another important feature is the capacity of DAPA to restore autophagic balance. In this study, the modulation of autophagy-related markers was obvious through the normalization of LC3-II levels and reduction of accumulated p62, which may reflect improved autophagic processing. Such reactivation thereby reduced mitochondrial ROS, improved organelle turnover, and indirectly diminished inflammasome activation. Restoration of autophagy represents a second protective step through which DAPA mitigates MTX-induced injury, in addition to its anti-inflammatory and anti-pyroptotic actions. It is important to note that LC3-II and p62 represent static markers of autophagy. While DAPA appears to restore autophagic processing, static markers cannot differentiate between enhanced autophagosome formation and blocked clearance, which warrants further validation *via* chloroquine/bafilomycin A1 flux assays (Gottlieb et al., 2015).

In spite of the DAPA’s promising hepatoprotective effect observed in this study, safety considerations are still to be considered. Previous reports indicated that DAPA may cause mild gastrointestinal disturbances such as diarrhea, which is also a known adverse effect of MTX (Klück et al., 2020; Wohlford et al., 2021). This potential overlap may suggest the need for further studies to evaluate the DAPA safety, especially when combined with some other drugs.

## 5 Conclusion

In conclusion, the present study demonstrates that DAPA can provide strong and multidimensional protection against MTX-induced hepatotoxicity in rats. This protection by DAPA is achieved through coordinated suppression of oxidative stress, inhibition of TLR4/MyD88/NF-κB signaling, and downregulation of NLRP3 inflammasome activation. Moreover, DAPA inhibits caspase-1/GSDMD-mediated pyroptosis and enhances autophagic activity. These actions altogether resulted in very good improvements in the tested parameters, including biochemical markers, histopathological findings, and molecular indices of hepatic injury. Known for its mechanistic specificity and previously demonstrated anti-inflammatory potential in preclinical and ongoing clinical studies, DAPA can be considered a good therapeutic candidate for mitigating MTX-associated liver toxicity. However, the clinical application of DAPA should be considered carefully since possible adverse effects, including diarrhea, may become aggravated when it is combined with methotrexate, which could affect patient tolerability and his quality of life. Therefore, further evaluation in chronic toxicity models, dose-escalation studies, and further translational clinical analyses will be essential to confirm its potential inclusion in MTX-containing treatment protocols.

## Data Availability

The original contributions presented in the study are included in the article/Supplementary Material, further inquiries can be directed to the corresponding author.
