# Supplementary material for: Dapansutrile mitigates methotrexate-induced hepatotoxicity in rats: roles of inflammation, oxidative stress, pyroptosis, and autophagy
Source: Front Pharmacol. 2026 Jun 23;17:1798557. doi: 10.3389/fphar.2026.1798557 (PMC13337402; doi:10.3389/fphar.2026.1798557)
Supplement: Supplementary file 1 [file Supplementaryfile1.docx]

Supplementary Material

**Supplementary Tables**

**Table S1. Biochemical Assay and ELISA Kits**

| Kit | Source | Cat. No. |
| --- | --- | --- |
| Serum alanine aminotransferase (ALT, SGPT) colorimetric kit | BioMed (Egypt) | GPT113100 |
| Serum alkaline phosphatase (ALP) colorimetric kit | BioMed (Egypt) | ALP101090 |
| Serum gamma-glutamyl transferase (GGT) kinetic kit | BioMed (Egypt) | GGT124030 |
| Malondialdehyde (MDA) colorimetric assay kit | Bio-Diagnostic (Giza, Egypt) | MD 25 28 |
| Reduced glutathione (GSH) colorimetric assay kit | Bio-Diagnostic (Giza, Egypt) | GR 25 10 |
| Total antioxidant capacity (TAC) assay kit | Bio-Diagnostic (Giza, Egypt) | TA 25 12 |
| Rat Toll-like receptor 4 (TLR4) ELISA kit | MyBioSource (San Diego, CA, USA) | MBS2024497 |
| Rat Myeloid differentiation factor 88 (MyD88) ELISA kit | MyBioSource (San Diego, CA, USA) | MBS2703631 |
| Rat tumor necrosis factor-α (TNF-α) ELISA kit | MyBioSource (San Diego, CA, USA) | MBS282960 |
| Rat interleukin-6 (IL-6) ELISA kit | MyBioSource (San Diego, CA, USA) | MBS2020158 |
| Rat interleukin-1β (IL-1β) ELISA kit | MyBioSource (San Diego, CA, USA) | MBS355368 |
| Rat interleukin-18 (IL-18) ELISA kit | Elabscience (Houston, TX, USA) | E-EL-R0567 |
| Rat NLR family pyrin domain-containing protein 3 (NLRP3) ELISA kit | MyBioSource (San Diego, CA, USA) | MBS7612469 |
| Rat apoptosis-associated speck-like protein containing CARD (ASC) ELISA kit | ELK Biotechnology, Sugar Land (Texas, USA) | ELK8931 |
| Rat gasdermin-D N-terminal (GSDMD-N) ELISA kit | ELK Biotechnology, Sugar Land (Texas, USA) | ELK7593 |

**Table S2. Antibodies Used for Immunohistochemistry and Western Blotting**

| Antibody | Molecular Weight | Host | Clonality | Dilution | Supplier | Cat. No. |  |
| --- | --- | --- | --- | --- | --- | --- | --- |
| NF-κB p65 | ~65 kDa | Rabbit | Polyclonal | 1:200 | ABclonal (Woburn, MA, USA) | A2547 | |
| Caspase-1 | ~45 kDa | Rabbit | Polyclonal | 1:200 | Servicebio (Wuhan, China) | GB11383 | |
| LC3A/LC3B | 14–16 kDa | Rabbit | Polyclonal | 1:1000 | Thermo Fisher Scientific (Rockford, IL, USA) | PA1-16931 | |
| p62 | ~62 kDa | Rabbit | Polyclonal | 1:1000 | Thermo Fisher Scientific (Rockford, IL, USA) | PA5-34781 | |
| β-Actin | 42 kDa | Mouse | Monoclonal | 1:5000 | Thermo Fisher Scientific (Rockford, IL, USA) | MA1-140 | |

**Table S3. Molecular Biology Reagents**

| Reagent | Supplier | Cat. No. |  |
| --- | --- | --- | --- |
| SV Total RNA Isolation System | Thermo Fisher Scientific (Rockford, IL, USA) | — |  |
| High-Capacity cDNA Reverse Transcription Kit | Thermo Fisher Scientific (Rockford, IL, USA) | — |  |
| Maxima SYBR Green qPCR Master Mix | Thermo Fisher Scientific (Rockford, IL, USA) | XNATRG-1KT | |
| StepOne™ Real-Time PCR System | Applied Biosystems (Carlsbad, CA, USA) | — |  |
| Ultravision One HRP Polymer Detection Kit | Thermo Fisher Scientific (Fremont, CA, USA) | — |  |
| Mayer’s hematoxylin | Standard laboratory grade | — |  |

**Table S4. Primer sequences used for quantitative real-time PCR**

| Gene | Forward Primer (5′→3′) | Reverse Primer (5′→3′) |
| --- | --- | --- |
| NLRP3 | GTGGAGATCCTAGGTTTCTCTG | CAGGATCTCATTCTCTTGGATC |
| NF-κB | TGCAGGCTCCTGTGCGAGTG | TCCGGTGGCGATCGTCTGTGT |
| GSDMD | CCAGCATGGAAGCCTTAGAG | CAGAGTCGAGCACCAGACAC |
| β-actin | CATTGCTGACAGGATGCAGAAGG | AGGGTGTAAAACGCAGCTCA |
